# Transactions of the American Dental Association

**Published:** 1887-02

**Authors:** 


### Bibliographical.

---

Transactions of the American Dental Association. —Twenty-sixth annual session, Niagara Falls, August, 1886. The S. S. White Dental Mfg. Co., Publishers.

This volume, compiled by the Publishing Committee, contains 191 pages and consists of synoptical reports of the papers and discussions of the session.

